# Ephedrine alkaloids-free Ephedra Herb extract: a safer alternative to ephedra with comparable analgesic, anticancer, and anti-influenza activities

**DOI:** 10.1007/s11418-016-0979-z

**Published:** 2016-03-04

**Authors:** Sumiko Hyuga, Masashi Hyuga, Naohiro Oshima, Takuro Maruyama, Hiroyuki Kamakura, Tadatoshi Yamashita, Morio Yoshimura, Yoshiaki Amakura, Takashi Hakamatsuka, Hiroshi Odaguchi, Yukihiro Goda, Toshihiko Hanawa

**Affiliations:** Oriental Medicine Research Center, Kitasato University, 5-9-1 Shirokane, Minato-ku, Tokyo, 108-8642 Japan; National Institute of Health Sciences, 1-18-1 Kamiyoga, Setagaya-ku, Tokyo, 158-8501 Japan; Department of Pharmaceutical Sciences, International University of Health and Welfare, 2600-1, Kitakanemaru, Ohtawara, Tochigi 324-8501 Japan; TOKIWA Phytochemical CO., Ltd., 158 Kinoko, Sakura-shi, Chiba 285-0801 Japan; Department of Pharmacognosy, College of Pharmaceutical Sciences, Matsuyama University, 4-2 Bunkyo-cho, Matsuyama, Ehime 790-8578 Japan

**Keywords:** Ephedra Herb, EFE, Kampo, c-Met, Analgesia, Influenza

## Abstract

It is generally accepted that the primary pharmacological activities and adverse effects of Ephedra Herb are caused by ephedrine alkaloids. Interestingly, our research shows that Ephedra Herb also has ephedrine alkaloid-independent pharmacological actions, such as c-MET inhibitory activity. This study describes the preparation of an ephedrine alkaloids-free Ephedra Herb extract (EFE) by ion-exchange column chromatography, as well as in vitro and in vivo evaluation of its pharmacological actions and toxicity. We confirmed that EFE suppressed hepatocyte growth factor (HGF)-induced cancer cell motility by preventing both HGF-induced phosphorylation of c-Met and its tyrosine kinase activity. We also investigated the analgesic effect of EFE. Although the analgesic effect of Ephedra Herb has traditionally been attributed to pseudoephedrine, oral administration of EFE reduced formalin-induced pain in a dose-dependent manner in mice. Furthermore, we confirmed the anti-influenza virus activity of EFE by showing inhibition of MDCK cell infection in a concentration-dependent manner. All assessments of toxicity, even after repeated oral administration, suggest that EFE would be a safer alternative to Ephedra Herb. The findings described here suggest that EFE has c-Met inhibitory action, analgesic effect, and anti-influenza activity, and that it is safer than Ephedra Herb extract itself. Therefore, EFE could be a useful pharmacological agent.

**Electronic supplementary material:**

The online version of this article (doi:10.1007/s11418-016-0979-z) contains supplementary material, which is available to authorized users.

## Introduction

Ephedra Herb is a crude drug containing ephedrine alkaloids, and is used in Japan as a component in many Kampo formulae, including maoto, kakkonto, shoseiryuto, and eppikajutsubuto. Ephedra Herb is defined in the sixteenth edition of the Japanese Pharmacopoeia (JP) as the terrestrial stem of *Ephedra sinica* Staf., *Ephedra intermedia* Schrenk et C.A. Meyer, or *Ephedra equisetina* Bunge (Ephedraceae), which have stems with ephedrine alkaloids (ephedrine and pseudoephedrine) content greater than 0.7 % [1]. Ephedra Herb has anti-inflammatory [2], analgesic, anti-influenza [3], and anti-metastatic effects [4]. However, because ephedrine alkaloids stimulate both sympathetic and parasympathetic nerves, Ephedra Herb has some adverse effects, including palpitations, hypertension, insomnia, and dysuria. The Food and Drug Administration (FDA) of the United States banned the sale of dietary supplements containing ephedra plants in 2004 because of health risks [5].

Since Professor Nagayoshi Nagai reported ephedrines to be the active constituents in Ephedra Herb [6], most of the pharmacological actions of Ephedra Herb have been attributed to ephedrine alkaloids, although the plant contains other constituents, such as phenolics and tannins [7]. Therefore, the adverse effects caused by ephedrine alkaloids are thought to be an unavoidable consequence associated with the pharmacological effects of Ephedra Herb.

Our previous research found that maoto, an Ephedra Herb-containing formulation, suppressed cancer metastasis by inhibiting cancer cell motility [8, 9] and prevented hepatocyte growth factor (HGF)-induced cancer cell motility by inhibiting phosphorylation of the c-Met receptor. Our studies confirmed that the c-Met inhibitory activity of maoto derives from Ephedra Herb, which impairs HGF-induced cancer cell motility by suppressing the HGF-c-Met signaling pathway through inhibition of c-Met tyrosine kinase activity [4]. HGF-c-Met signaling regulates several cellular processes, including cell proliferation, invasion, scattering, survival, and angiogenesis. Dysregulation of HGF-c-Met signaling promotes tumor formation, growth, progression, metastasis, and therapeutic resistance [10, 11]. Therefore, Ephedra Herb may have applications in cancer therapy as a novel c-Met inhibitor. Moreover, we have discovered that Ephedra Herb contains herbacetin 7-*O*-neohesperidoside and herbacetin 7-*O*-glucoside [12]. Herbacetin, the aglycone of these herbacetin-glycosides, inhibits HGF-induced cell migration and phosphorylation of c-Met [13]. These findings suggest that herbacetin-glycosides are bioactive constituents of Ephedra Herb that may be responsible for its pharmacological actions not mediated by ephedrine alkaloids. However, the c-Met inhibitory activity of Ephedra Herb extract cannot be explained by herbacetin-glycosides alone, because the herbacetin-glycoside content in Ephedra Herb extract is less than 0.1 % [14]. Moreover, we confirmed that ephedrine had no effect on HGF-c-Met signaling [15]. Therefore, we predicted that c-Met inhibitory activity may be produced by the non-alkaloidal fraction of Ephedra Herb extract, which contains herbacetin-glycosides and other bioactive molecules that produce synergistic effects. The non-alkaloidal fraction of Ephedra Herb is useful for cancer patients, because adverse effects caused by ephedrine alkaloids are avoided.

We utilized ion-exchange column chromatography to eliminate ephedrine alkaloids from Ephedra Herb extract, resulting in ephedrine alkaloids-free Ephedra Herb extract (EFE) [14]. In this study, we report the pharmacological and toxicological properties of EFE.

## Materials and methods

### Materials

Ephedrine was purchased from Dainippon Pharma Co., Ltd. (Tokyo, Japan). Methylephedrine and pseudoephedrine were purchased from Alps Pharmaceutical Ind. Co. Ltd. (Gifu, Japan). Norephedrine was purchased from Tokyo Chemical Industry Co. Ltd. (Tokyo, Japan). 6-Methoxykynurenic acid was purchased from Chemicia Scientific, LLC (San Diego, CA, USA). *trans*-Cinnamic acid was purchased from Wako Co. (Tokyo, Japan). 6-Hydroxykynurenic acid was synthesized from 6-methoxykynurenic acid [14]. Syringin, kaempferol 3-*O*-rhamnoside 7-*O*-glucoside, and isovitexin 2″-*O*-rhamnoside were isolated from Ephedra Herb [12]. The methanol and water used for liquid chromatography coupled with photodiode array detection (LC-PDA) analysis were LC grade.

Recombinant human HGF (purity ≥98 % by SDS-PAGE and HPLC analysis) was purchased from PeproTech Inc. (Rocky Hill, NJ, USA). SU11274 (purity ≥98 % by HPLC analysis) was purchased from Sigma-Aldrich (St Louis, MO, USA). The antibodies (Abs) used were as follows: anti-p-Met (Tyr1234/1235) monoclonal Ab (mAb) (CST#3077), anti-Met mAb (CST#8198), anti-glyceraldehyde-3-phosphate dehydrogenase (GAPD) mAb (CST#2118), and horseradish peroxidase (HRP)-labeled anti-rabbit IgG Ab (CST#7074); all were obtained from Cell Signaling Technology Japan, K.K. (Tokyo, Japan).

### Preparation of EFE and Ephedra Herb extract

Preparation of EFE and Ephedra Herb extract was carried out as described by Oshima et al. [14]. Ephedra Herb (200 g, *E. sinica*, Japanese pharmacopoeia grade) was added to water (2000 ml), extracted at 95 °C for 1 h, and filtered, after which the residue was washed with water (200 ml). The extract was centrifuged at 1800*g* for 10 min, after which half of the supernatant was concentrated under reduced pressure to obtain Ephedra Herb extract (14.1 g), while the other half was passed directly through DIAION™ SK-1B ion-exchange resin (100 ml) which was treated with 1 M HCl (30 ml) and water (100 ml) prior to use, then washed with water (100 ml). The unadsorbed fraction (1100 ml) was adjusted to pH 5 using 5 % NaHCO_3_ aq. (60 ml), and the solution was then evaporated under reduced pressure to obtain EFE (11.8 g).

### LC-PDA analysis of Ephedra Herb extract and EFE

One milliliter of methanol was added to 50 mg samples of Ephedra Herb extract and EFE, which were exposed to ultrasonic waves for 30 min and centrifuged. The supernatants were filtered through 0.45-µm membrane filters, after which 20 µl of each sample was subjected to LC-PDA analysis.

LC-PDA analysis was performed using an LC-10A HPLC system consisting of an SCL-10Avp system controller, an LC-10ATvp pump, a DGU-12A degasser, an SIL-10A auto injector, a CTO-10Avp column oven, and an SPD-M10Avp photodiode array detector equipped with a semi-micro cell (Shimadzu Inc., Tokyo, Japan). An Inertsil ODS-3 column (4.6 × 150 mm, 5 µm) from GL Sciences (Tokyo, Japan) was used for the separation. The column was maintained at 40 °C in the column oven. The mobile phase consisted of 0.1 % formic acid in water (A) and 0.1 % formic acid in methanol (B). The flow rate was 1.0 ml/min. The mobile phase gradient was adjusted as follows: 5 % B (0–10 min), 5–75 % B (10–70 min), 75–100 % B (70–80 min), 100 % B (80–90 min).

### Cell lines and culture

MDA-MB-231 human breast cancer cells were obtained from the American Type Culture Collection (Manassas, VA, USA) and cultured in DMEM (Sigma-Aldrich, St. Louis, MA, USA) containing 10 % fetal calf serum (FCS) (Invitrogen Corp., Carlsbad, CA, USA) at 37 °C in an atmosphere containing 5 % CO_2_.

### Trans-well migration assay

MDA-MB-231 cells (5 × 10^4^ cells/100 µl) were suspended in 100 μl DMEM containing EFE (10, 20, or 40 μg/ml), Ephedra Herb extract (40 μg/ml), or SU11274 (5 μM). The cells were poured into the upper well of a trans-well permeable support system (Corning Inc., Acton, MA, USA). DMEM (600 µl) containing 50 ng/ml HGF was added to the lower well of the trans-well system, which was incubated for 20 h at 37 °C. Finally, the number of cells that had migrated from the upper layer to the lower well was counted.

### Cell viability

MDA-MB-231 cells (5 × 10^4^ cells/100 μl) were suspended in 100 μl of 10 % FCS-DMEM containing 40 μg/ml EFE, 40 μg/ml Ephedra Herb extract, or 5 μM SU11274. After 20 h, 10 μl of Cell Counting Kit-8 solution (Dojindo, Kumamoto, Japan) was added to each sample, after which the resulting mixture was incubated at 37 °C for 2 h. Formazan absorbance (450 nm) was quantified using an iMark microplate reader (Bio-Rad Laboratories, Hercules, CA, USA).

### Detection of phosphorylated c-Met (p-Met), c-Met, and GAPDH

MDA-MB-231 cells (2 × 10^6^ cells/4 ml) were incubated in 4 ml of 10 % FCS-DMEM for 48 h, washed three times with DMEM, and incubated in 4 ml DMEM for 24 h. After the cells were washed three times with DMEM, they were incubated for 15 min at 37 °C in 4 ml DMEM or DMEM containing 50 ng/ml of HGF with or without 0.5, 1, or 10 μg/ml EFE, 10 μg/ml Ephedra Herb extract, or 5 μM SU11274. After the cells were washed three times with cold PBS without Ca^2+^ and Mg^2+^ (PBS(-)) they were treated with 1 ml Complete Lysis-M with phosphatase inhibitor (Roche Diagnostics Co., Indianapolis, IN, USA) for 5 min on an ice bath. The lysates were collected and centrifuged, after which the supernatants were incubated with 5× sodium dodecyl sulfate (SDS) loading buffer for 5 min at 95 °C. The lysates were separated by SDS–polyacrylamide gel electrophoresis (PAGE) and electro-transferred to a polyvinylidene difluoride (PVDF) membrane. The membrane was blocked at room temperature for 1 h with 5 % non-fat dry milk in Tris-buffered saline (10 mM Tris–HCl, pH 7.5, 100 mM NaCl) containing 0.1 % Tween 20 (TBS-T). After the membrane was washed with TBS-T, it was incubated with the anti-p-Met (Tyr1234/1235) mAb (CST#3077), anti-Met mAb (CST #8198), or anti-GAPDH Ab (SC-25778) overnight at 4 °C and washed with TBS-T. Horseradish peroxidase-labeled anti-rabbit IgG Ab (CST#7074) was applied for 1 h at room temperature, after which the membranes were washed with TBS-T. The Abs were detected with an enhanced chemiluminescent (ECL) reaction (GE Healthcare Japan, Tokyo, Japan) and imaged using an ImageQuant Las 4000 mini system (GE Healthcare Japan).

### Measurement of c-Met tyrosine-kinase activity and determination of IC_50_ values

Met kinase activity was measured using the ADP-Glo kinase assay kit (Promega, Madison, WI, USA) according to the manufacturer’s instructions. Briefly, 10 μl of a reaction mixture containing 2 μg/ml of recombinant Met kinase domain, 0.2 μg/ml poly(E4Y1), and 10 μM ATP was incubated with Ephedra Herb extract or EFE at room temperature for 60 min. The kinase reactions were terminated by the addition of 10 μl ADP-Glo reagent, after which the resulting mixture was incubated for 40 min at room temperature. Next, 20 μl of Kinase Detection Reagent was added, after which the mixture was incubated for 30 min at room temperature. Luminescence was measured with an EnSpire multi-plate reader (Perkin Elmer, Foster City, CA, USA). The experiments were repeated three times. Each IC_50_ was calculated using a four-parameter logistic model (Prism 5.0, GraphPad Software, San Diego, CA, USA).

### Formalin test

ICR male mice (5 weeks of age, 8 mice per group) were orally administered water, 350 mg/kg EFE, or 700 mg/kg Ephedra Herb extract for 3 days. On the third day, paw-licking was induced in the mice by intraplantar injection of 20 μl of 2.5 % formalin 6 h after extract/water administration. After the injection, the mice were individually placed into a glass cage, in which the amount of time that the animal spent licking the injected paw was measured as an indicator of pain. Paw-licking was recorded for 30 min in two phases, the first phase (0–5 min) and second phase (15–30 min).

The protocol for animal experiments was approved by the Ethics Review Committee for Animal Experimentation of the National Institute of Health Sciences.

### Evaluation of anti-influenza activity

Madin–Darby canine kidney (MDCK) cells (3 × 10^4^ cells/100 μl) were incubated in 100 μl of 10 % FCS-minimal essential medium (MEM) in a 96-well plate for 24 h and washed with MEM. Next, the cells were incubated for 72 h at 37 °C in 100 μl of MEM or MEM containing a twofold serial dilution of 10 μM oseltamivir, 50 μg/ml EFE, or 50 μg/ml Ephedra Herb extract with or without 100 TCID_50_ of influenza virus A/WSN/33(H1N1). Living cells were then stained with crystal violet, after which the absorbance (560 nm) of each cell sample was quantified using a microplate reader. These experiments were performed externally by AVSS Corporation. Each IC_50_ was calculated using a four-parameter logistic model (Prism 5.0, GraphPad Software).

### Repeated oral dose toxicity assessment

Special pathogen-free ICR mice (Crl:CD1) (5 weeks old) were obtained from Charles River Laboratories (Boston, MA, USA). The mice were kept in a laboratory animal facility with temperature and relative humidity maintained at 20–26 °C and 30–70 %, respectively, a 12 h-light–dark cycle, and 8–10 air charges per hour. The mice were housed in polycarbonate cages and offered CE-2 pellet feed (Nippon Formula Feed Mfg. Co., Ltd., Ehime, Japan) and groundwater that was disinfected with 0.5 % chlorine and filtered through a 5-μm filter. The mice were acclimated for 7 days before the start of the study. The mice were grouped into three groups: water, EFE, or Ephedra Herb extract. Each group included five male mice and five female mice.

The dosages of EFE and Ephedra Herb extract were converted to 50-fold of the human maximum dose of Ephedra Herb extract, equivalent to 6 g of cut crude drug. The doses of EFE and Ephedra Herb extract were 632 mg/kg/day and 755 mg/kg/day, respectively. The mice were orally administered water, EFE, or Ephedra Herb extract once per day for 14 days. Clinical signs and mortality were assessed several times per day. Body weight, food consumption, and water consumption were measured twice per week throughout the experiment. After 14 days, all mice were anesthetized by isoflurane inhalation, after which blood samples were collected from the abdominal aorta.

Daiich Negishi Clinical Laboratory, Inc. assessed the following hematological parameters: red blood cell count (RBC), hematocrit (Ht), hemoglobin concentration (Hb), platelet count (PLT), white blood cell count (WBC), and WBC differential count (stab neutrophils, segmented neutrophils, lymphocytes, and monocytes). Serum biochemical parameters were measured by the Nagahama Institute for Biochemical Science at Oriental Yeast Co., Ltd. The selected serum biochemical parameters were albumin (ALB), aspartate aminotransferase (ALT), alanine aminotransferase (ALT), alkaline phosphatase (ALP), lactate dehydrogenase (LDH), leucine aminopeptidase (LAP), gamma-glutamyl transpeptidase (γ-GT), and total bilirubin (T-BIL).

After the collection of the blood samples, the organs were harvested from each mouse and weighed. Colon weight was measured after washing out the colon contents with saline solution.

### Statistical analysis

All data are expressed as mean ± standard deviation (SD). Data were analyzed by ANOVA. Significant differences between the control and treatment groups were determined by Student’s *t* test, Dunnett’s test, and Tukey’s test using GraphPad Prism 5J software (MDF Co., Ltd., Tokyo, Japan). *p* < 0.05 was considered statistically significant.

## Results

### 3D-HPLC analysis of Ephedra Herb extract and EFE

**Fig. 1:**
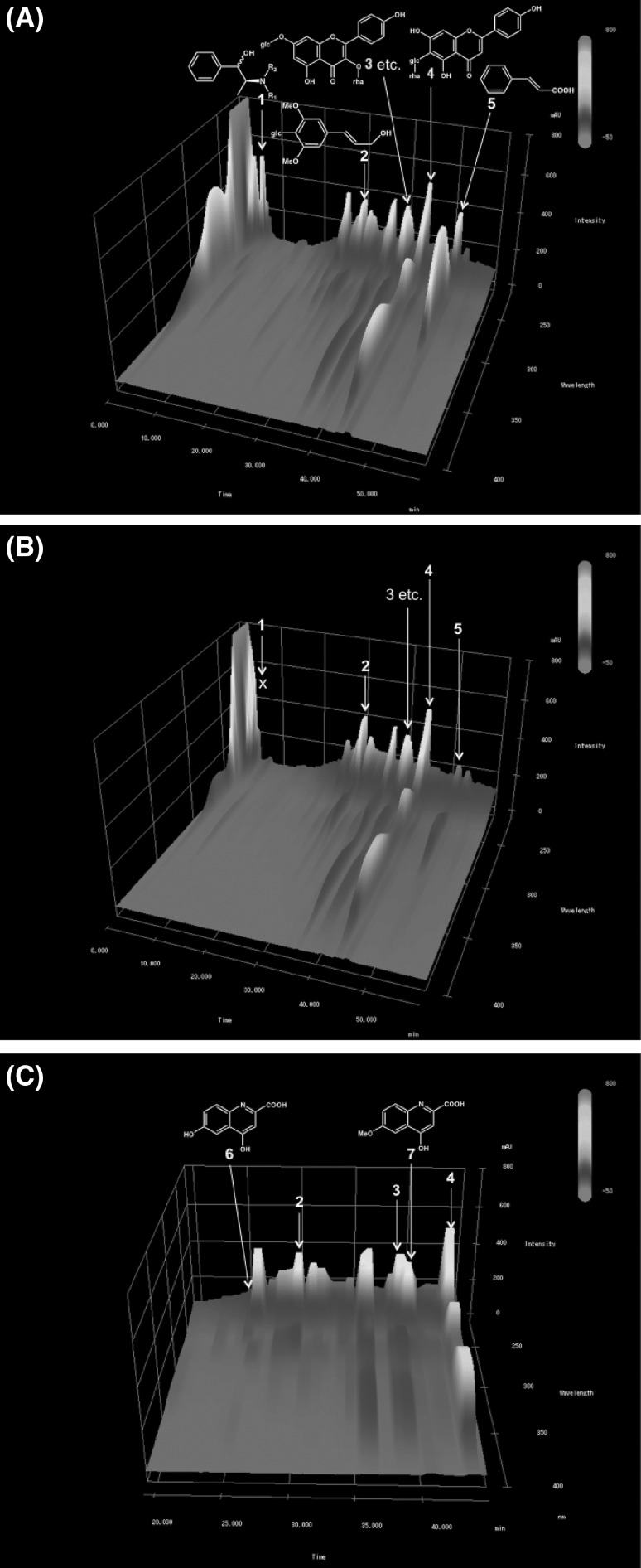
3D-HPLC profile of Ephedra Herb extract and EFE. **a** Ephedra Herb extract; **b** EFE; **c** zoom at 20–45 min in Ephedra Herb extract. *1* ephedrine alkaloids; *2* syringin; *3* kaempferol 3-*O*-rhamnoside 7-*O*-glucoside; *4* isovitexin 2″-*O*-rhamnoside; *5* cinnamic acid; *6* 6-hydroxykynurenic acid; *7* 6-methoxykynurenic acid

Ephedra Herb extract and EFE displayed similar chromatographic profiles, but several peaks were absent in that of EFE (Fig. 1a, b). The retention times and UV spectra of the Ephedra Herb extract standard revealed the presence of ephedrine alkaloids (ephedrine, pseudoephedrine, norephedrine, and methylephedrine), 6-hydroxykynurenic acid, syringin, kaempferol 3-*O*-rhamnoside 7-*O*-glucoside, 6-methoxykynurenic acid, isovitexin 2″-*O*-rhamnoside, and cinnamic acid (Fig. 1a, c). However, ephedrine alkaloids, 6-hydroxykynurenic acid, and 6-methoxykynurenic acid were not present in the EFE chromatogram (Fig. 1a, b) [14].

### Inhibitory effect of EFE on HGF-induced motility of MDA-MB-231 cells

**Fig. 2:**
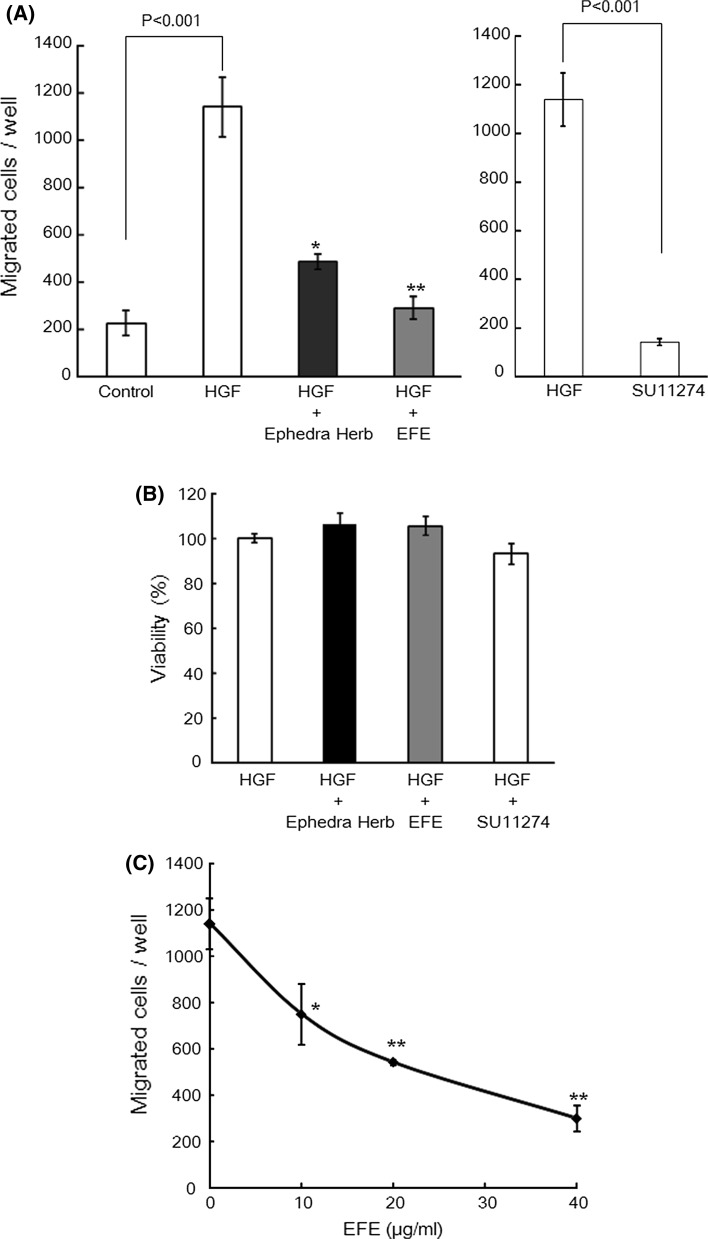
Effects of EFE, Ephedra Herb extract, and c-Met inhibitor SU11274 on HGF-induced motility and viability of MDA-MB-231 cells. **a** MDA-MB-231 cells (5 × 10^4^ cells) were suspended in DMEM with or without 40 μg/ml Ephedra Herb, 40 μg/ml EFE, or 5 μM SU11274, and poured into the upper well of the trans-well system. The lower well of the trans-well system contained 600 µL of DMEM containing 50 ng/ml HGF. After 20 h, the cells that had migrated into the lower well were counted. Each assay was performed in triplicate. The *error bars* represent standard deviation. Statistical significance was determined by Dunnett’s test. **p < 0.001 vs. the number of cells that migrated following HGF stimulation. **b** 5 × 10^4^ cells were suspended in 100 μl of DMEM containing 50 ng/ml HGF with or without 40 μg/ml Ephedra Herb, 40 μg/ml EFE, or 5 μM SU11274. After 20 h, cell viability was analyzed using the Cell Counting Kit-8 as described in the “Materials and methods” section. Viability (%) is expressed as (absorbance of cells in DMEM containing HGF and crude drug extract/absorbance of cells in DMEM containing HGF) × 100. Each assay was performed in triplicate. The *error bars* represent standard deviation. Statistical significance was determined by Dunnett’s test. **c** 5 × 10^4^ cells were suspended in DMEM with 0, 10, 20, or 40 μg/ml EFE and poured into the upper well of the trans-well system. The lower well of the trans-well system contained DMEM with 50 ng/ml HGF. After 20 h, the cells that had migrated to the lower well were counted. Each assay was performed in triplicate. The *error bars* represent standard deviation. Statistical significance was determined by Dunnett’s test. *p < 0.01, or **p < 0.001 vs. the number of cells that migrated without EFE exposure

We confirmed the inhibitory effect of EFE on HGF-induced motility of MDA-MB-231 cells using a trans-well permeable support system. HGF (50 ng/ml) significantly induced MDA-MB-231 cell motility; however, this effect was inhibited by 5 μM SU11274, a c-Met-specific inhibitor (Fig. 2a). We previously reported that 40 μg/ml Ephedra Herb extract suppressed the HGF-induced migration of MDA-MB-231 cells [4], so the same concentration was used in this study. The results show that both EFE and Ephedra Herb extract significantly suppressed the HGF-induced motility at a concentration of 40 μg/ml (Fig. 2a).

We also examined the effects of Ephedra Herb extract and EFE on the viability of MDA-MB-231 cells and found that the extracts had no effect on cell viability (Fig. 2b). This indicates that the inhibitory activities of these extracts on HGF-induced motility are independent of cytotoxicity.

Subsequently, we investigated the effects of various concentrations of EFE on HGF-induced migration of MDA-MB-231 cells. We found that EFE significantly inhibited the HGF-induced motility of MDA-MB-231 cells in a concentration-dependent manner (Fig. 2c), thus confirming that EFE possesses inhibitory activity against HGF-induced cancer cell motility.

### Inhibitory effect of EFE on HGF-induced c-Met phosphorylation and tyrosine kinase activity

**Fig. 3:**
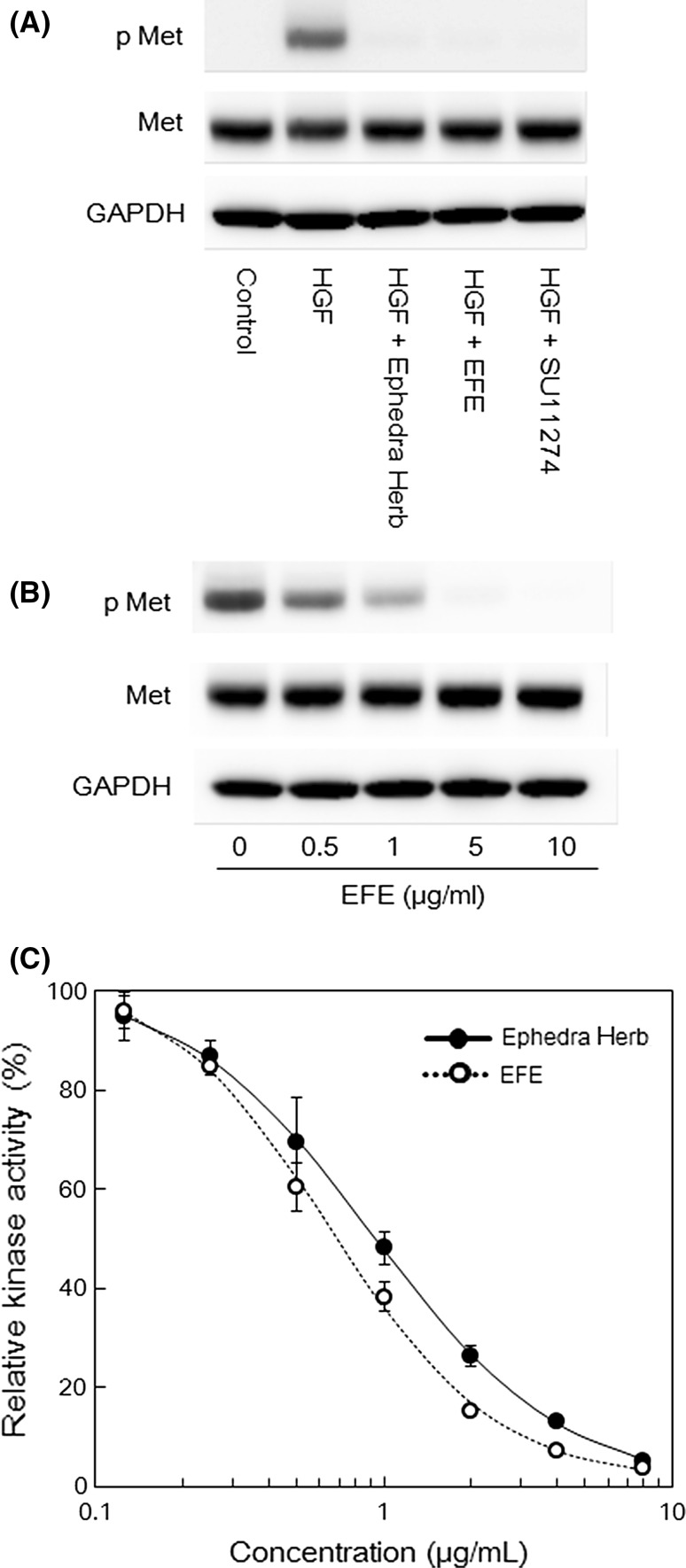
Effects of EFE, Ephedra Herb extract, and SU11274 on HGF-induced phosphorylation of c-Met, and effects of EFE and Ephedra Herb extract on the tyrosine-kinase activity of c-Met. **a** MDA-MB-231 cells were incubated in DMEM, DMEM containing 50 ng/ml HGF, or DMEM containing 50 ng/ml HGF with 10 μg/ml EFE, 10 μg/ml Ephedra Herb extract, or 5 μM SU11274 for 15 min at 37 °C. Tyrosine phosphorylation of c-Met was determined by immunoprecipitation and Western blot analysis. **b** MDA-MB-231 cells were incubated in DMEM containing 50 ng/ml of HGF with 0, 0.5, 1, 5, or 10 μg/ml of EFE for 15 min at 37 °C. The level of tyrosine phosphorylation of c-Met in the cells was determined by immunoprecipitation and Western blot analysis. **c** The kinase activity of c-Met was measured using the ProfilerPro Kit. A recombinant c-Met kinase domain was pre-incubated with and without a twofold serial dilution of 8 μg/ml EFE or Ephedra Herb extract at 28 °C for 15 min. The fluorescence-labeled peptide substrate, 1.5 μM 5-carboxyfluorescein-EAIYAAPFAKKK-NH_2_, and 79.5 μM ATP were added, followed by incubation at 28 °C for 90 min. The kinase reactions were terminated by the addition of 3 mM EDTA. Phosphorylated peptides were separated from substrate peptides and quantified using a LabChip 3000

HGF binding activates c-Met, which initiates receptor dimerization and auto-phosphorylation of tyrosine residues, propagating downstream signals. Accordingly, we confirmed the inhibitory effect of EFE on HGF-induced phosphorylation of c-Met in MDA-MB-231 cells. Tyrosine phosphorylation of c-Met was induced by HGF (50 ng/ml) and inhibited by 5 μM SU11274 (Fig. 3a). An Ephedra Herb extract concentration of 10 μg/ml was used in this study because we demonstrated in a previous study that 10 μg/ml Ephedra Herb extract suppressed HGF-induced phosphorylation of c-Met [4]. No phosphorylation of c-Met was observed following the addition of 50 ng/ml HGF with 10 μg/ml EFE (Fig. 3a). Moreover, we investigated the effects of various concentrations of EFE on the HGF-induced phosphorylation of c-Met. EFE prevented the HGF-induced phosphorylation of c-Met in a concentration-dependent manner (Fig. 3b). We also investigated the inhibitory activity of EFE on tyrosine kinase activity of c-Met. Ephedra Herb extract and EFE produced concentration-dependent inhibition of the tyrosine kinase activity of c-Met (Fig. 3c). The IC_50_ of Ephedra Herb extract and EFE were 0.887 and 0.530 μg/ml, respectively.

These results suggest that EFE suppresses the HGF-induced motility by inhibiting the phosphorylation of c-Met though the prevention of tyrosine kinase activity. In addition, the c-Met inhibitory activity of Ephedra Herb was confirmed to be independent of ephedrine alkaloids.

### Effect of EFE on formalin-induced pain in mice

**Fig. 4:**
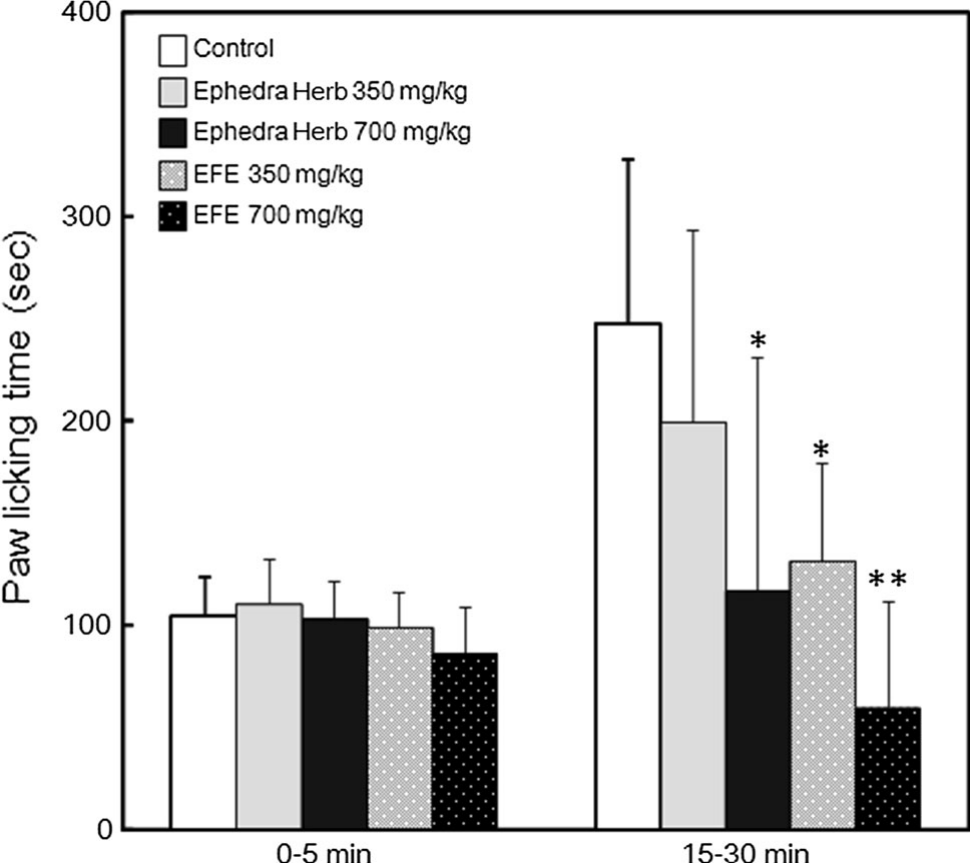
Effects of EFE and Ephedra Herb extract on formalin-induced pain. ICR mice were treated orally with water, 350 mg/kg EFE, 700 mg/kg EFE, or Ephedra Herb extract for 3 days. On the third day of treatment, formalin tests were performed 6 h after drug or placebo administration. The amount of time that each animal spent licking the injection paw was recorded for 30 min in two phases, the first (0–5 min) and second (15–30 min) phases. Statistical significance was determined by Dunnett’s test. *p < 0.05 or **p < 0.01 vs. control

The analgesic effect of Ephedra Herb has been traditionally believed to be mediated by pseudoephedrine [2, 16], but we recently found that herbacetin, a component of Ephedra Herb, suppressed the formalin-induced pain [17]. Therefore, we examined the analgesic effect of EFE using the formalin test. Ephedra Herb extract and EFE showed no effects during the first phase of the formalin test. Ephedra Herb extract and EFE reduced paw-licking time in a dose-dependent manner during the second phase of the formalin test. The paw-licking time during the second phase of the formalin test was significantly decreased by oral administration of 700 mg/kg Ephedra Herb extract, 350 mg/kg EFE, and 700 mg/kg EFE (Fig. 4). These results reveal that EFE possessed the analgesic action.

### Effect of EFE on influenza virus infection in MDCK cells

**Fig. 5:**
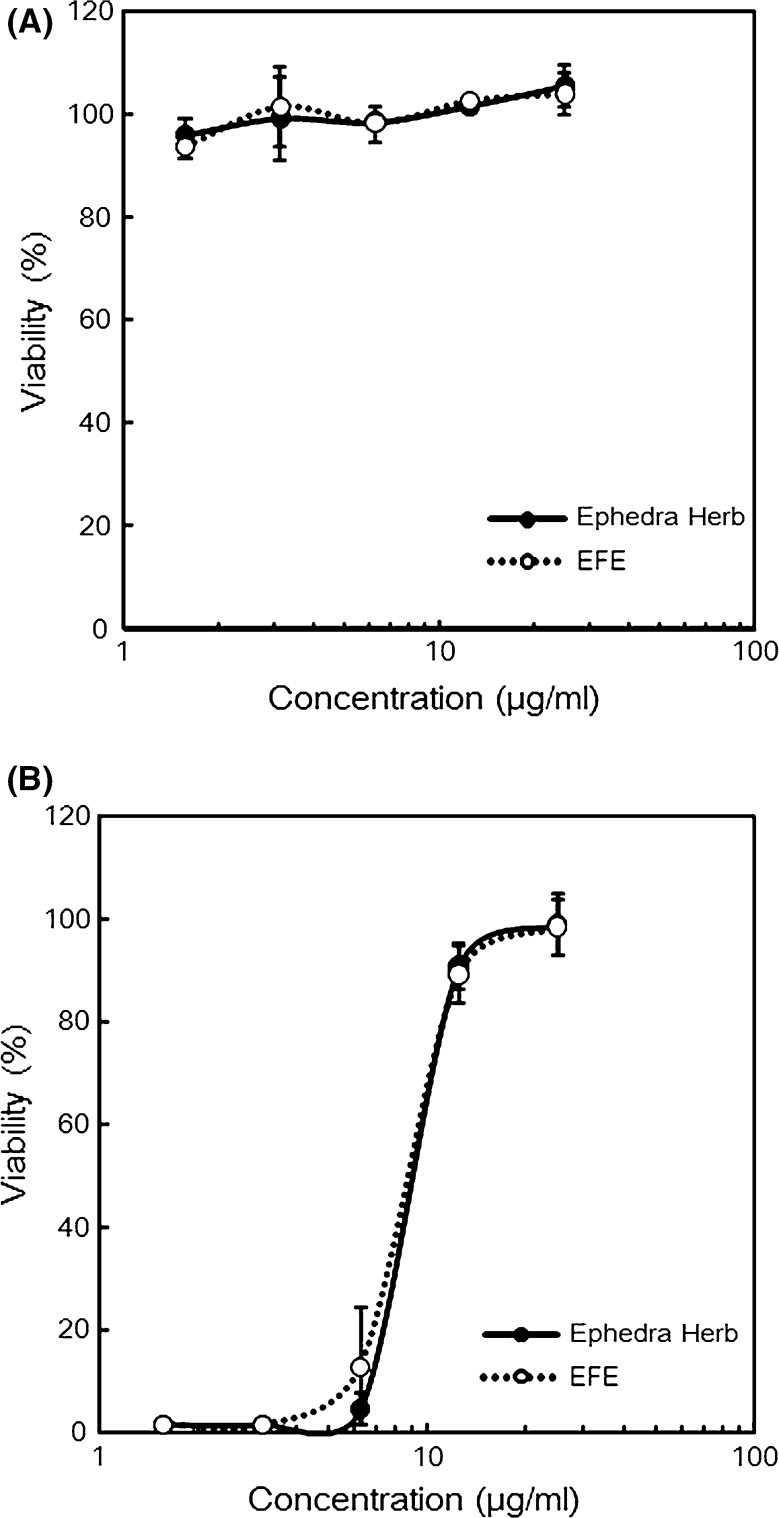
Effects of EFE and Ephedra Herb extract on influenza virus infection in MDCK cells. MDCK cells (3 × 10^4^ cells) were incubated in 100 μl of 10 % FCS-MEM in a 96-well plate for 24 h, and then washed with MEM. They were incubated for 72 h at 37 °C in 100 μl of MEM or MEM containing a twofold serial dilution of 50 μg/ml EFE or 50 μg/ml Ephedra Herb extract with (**b**) or without (**a**) 100 TCID_50_ of influenza virus A/WSN/33(H1N1). Next, living cells were stained with crystal violet and the absorbance (560 nm) of each sample was quantified using a microplate reader

Ephedra Herb has been reported to possess anti-influenza activity [3]. Therefore, we examined the effect of EFE on the survival rate of MDCK cells infected with influenza virus A/WSN/33(H1N1). Oseltamivir, an anti-influenza drug, suppressed influenza virus infection in MDCK cells in a concentration-dependent manner without causing cytotoxicity (See Supplemental Fig. 1). The IC_50_ of oseltamivir was 3.49 μM. Neither Ephedra Herb extract nor EFE affected MDCK cell viability (Fig. 5a), whereas Ephedra Herb extract and EFE prevented cell death caused by influenza virus infection in a concentration-dependent manner (Fig. 5b). The IC_50_ values of Ephedra Herb extract and EFE were 8.6 μg/ml and 8.3 μg/ml, respectively. These results indicate that EFE retains the anti-influenza activity of Ephedra Herb, indicating that this activity is not mediated by ephedrine alkaloids.

### Safety assessment of EFE

We evaluated the safety of EFE in comparison with that of Ephedra Herb extract and water through a repeated-dose toxicity study. Extract-related death and abnormal clinical signs were not observed in mice treated with EFE or Ephedra Herb extract during the testing period. After 2 weeks, gross abnormalities were not observed in any of the treated mice, and there were no significant differences in mice weights between the three groups (Table 1). However, the colon weights of the male mice subjected to oral administration of EFE or Ephedra Herb extract were significantly lower than those of male mice treated with water, although the reduction was moderate. Differences in the colon weights of the groups of female mice were not significant. There was no significant difference in the weight of any other tissue among the groups (Table 1).

**Table 1:** Body and tissue weight

Sex	Group		Body weight (g)	Thymus (mg)	Heart (mg)	Lung (mg)	Liver (mg)	Adrenal gland (mg)	Kidney (right) (mg)	Kidney (left) (mg)	Spleen (mg)	Stomach (mg)	Small intestine (mg)	Cecum (mg)	Colon (mg)
Male	Water	AVG	32.3	63.7	158.2	177.5	1939.0	7.2	262.7	244.4	97.8	256.1	1915.1	153.2	382.3
		SD	1.5	14.6	13.9	34.0	186.6	1.9	37.7	35.0	16.9	28.3	279.7	14.9	14.6
	EFE	AVG	30.9	64.9	152.4	171.2	1777.8	7.3	237.1	239.3	90.5	231.0	1741.5	133.5	310.4**
		SD	2.9	22.2	14.7	17.7	169.4	1.6	27.0	24.5	13.6	28.1	318.3	16.2	35.6
	Ephedra Herb	AVG	30.8	43.6	151.7	195.8	1674.2	5.8	243.9	238.3	90.3	252.3	1912.2	161.7	326.9*
		SD	1.2	6.3	17.8	43.2	196.9	0.8	22.8	21.8	17.7	22.7	235.0	33.1	22.5
Female	Water	AVG	26.3	67.0	140.5	154.3	1350.3	10.0	167.3	157.2	88.1	205.7	1397.0	121.2	338.6
		SD	2.3	46.4	14.9	10.2	162.3	1.2	12.3	14.0	13.0	18.2	144.3	14.3	91.3
	Ephedra Herb	AVG	26.1	55.6	135.9	158.8	1208.8	10.3	159.6	154.4	93.1	215.9	1202.8	109.8	282.1
		SD	1.5	7.7	9.4	10.8	121.4	1.1	11.4	10.9	8.1	15.4	92.2	13.2	45.3
	EFE	AVG	26.5	54.2	142.5	161.6	1223.6	9.8	171.8	172.1	113.9	216.8	1324.4	140.8	274.1
		SD	2.8	13.2	24.1	13.9	215.5	2.3	29.2	35.1	42.2	26.7	217.9	25.5	22.1

Sex	Group		Testis (mg)	Vesicular gland (mg)	Vas deferens (mg)	Epididymis (mg)	Ovary (mg)	Uterus (mg)
Male	Water	AVG	199.4	170.5	30.8	78.3	–	–
		SD	20.9	75.5	7.0	12.3	–	–
	EFE	AVG	199.7	149.3	26.1	77.0	–	–
		SD	24.3	37.5	2.3	6.3	–	–
	Ephedra Herb	AVG	198.6	126.0	25.7	74.4	–	–
		SD	13.0	36.8	3.9	5.8	–	–
Female	Water	AVG	–	–	–	–	13.1	125.9
		SD	–	–	–	–	1.0	43.7
	Ephedra Herb	AVG	–	–	–	–	12.4	167.1
		SD	–	–	–	–	2.4	66.9
	EFE	AVG	–	–	–	–	13.8	145.4
		SD	–	–	–	–	6.2	53.0

* *P* < 0.05 and ** P < 0.01 vs. mice administered water, by Tukey’s test

Serum biochemistry and hematological data are shown in Tables 2 and 3, respectively. The groups showed no significant differences in serum biochemistry parameters (Table 2). The PLT of male mice taking Ephedra Herb extract was significantly higher than that of the water group, but there was no significant difference between the PLT of the male EFE-treated group and that of the male water-treated group (Table 3). The WBC of male mice treated with Ephedra Herb extract was significantly lower than of the water male group, but there was no significant difference between the WBC of the male EFE-treated group and that of the male water-treated group (Table 3). The groups of female mice showed no significant differences in any hematological parameter. These results indicate that EFE may be less toxic than Ephedra Herb extract and could represent a safer alternative.

**Table 2:** Serum biochemical values

Sex	Parameter	ALB (g/dl)	AST (IU/l)	ALT (IU/l)	ALP (IU/l)	LDH (IU/l)	LAP (IU/l)	γ-GT (IU/1)	T-BIL (mg/dl)
	Group	AVG ± SD	AVG ± SD	AVG ± SD	AVG ± SD	AVG ± SD	AVG ± SD	AVG ± SD	AVG ± SD
Male	Water	2.86 ± 0.13	51.8 ± 10.9	34.2 ± 10.8	292.6 ± 51.6	597.2 ± 447.8	58.4 ± 3.8	N.D.	0.08 ± 0.014
	EFE	2.96 ± 0.17	50.2 ± 8.3	26.4 ± 2.7	280.2 ± 46.3	765.0 ± 233.2	53.8 ± 3.2	N.D.	0.10 ± 0.024
	Ephedra Herb	2.90 ± 0.10	53.4 ± 4.7	29.0 ± 4.3	384.6 ± 191.2	727.0 ± 251.9	60.6 ± 2.1	N.D.	0.08 ± 0.008
Female	Water	3.12 ± 0.08	53.4 ± 10.7	25.0 ± 3.9	332.8 ± 38.8	435.6 ± 214.0	54.2 ± 4.7	N.D.	0.08 ± 0.015
	EFE	3.06 ± 0.18	55.0 ± 9.7	21.6 ± 2.6	361.2 ± 50.6	645.8 ± 224.5	55.0 ± 3.2	N.D.	0.10 ± 0.004
	Ephedra Herb	3.24 ± 0.19	57.8 ± 6.9	21.6 ± 6.1	332.4 ± 60.1	644.2 ± 91.4	58.6 ± 7.2	N.D.	0.08 ± 0.025

*N.D* not detectable

**Table 3:** Haematological values

Sex	Parameter	RBC (10,000/μl)	Hb (g/dl)	Ht (%)	PLT (10000/μl)	WBC (1000/μl)	Neutro (Stab) %	Neutro (Seg)%	Lympho (%)	Mono (%)
	Group	AVG ± SD	AVG ± SD	AVG ± SD	AVG ± SD	AVG ± SD	AVG ± SD	AVG ± SD	AVG ± SD	AVG ± SD
Male	Water	926 ± 25	15.1 ± 0.6	50.0 ± 1.6	49.8 ± 8.4	2.27 ± 0.94	2.2 ± 0.4	33.4 ± 9.4	59.6 ± 9.0	4.8 ± 1.1
	EFE	964 ± 21	15.6 ± 0.6	49.6 ± 1.6	48.2 ± 8.6	1.27 ± 0.63	2.6 ± 0.9	18.8 ± 12.9	74.2 ± 13.8	4.4 ± 2.1
	Ephedra Herb	976 ± 40	15.2 ± 0.8	49.5 ± 1.8	65.2 ± 10.4*	0.90 ± 0.17*	2.4 ± 0.9	23.6 ± 12.4	70.4 ± 13.1	3.6 ± 2.2
Female	Water	947 ± 48	15.5 ± 1.4	49.6 ± 3.5	44.2 ± 12.3	2.49 ± 1.4	2.0 ± 1.0	13.0 ± 11.0	80.0 ± 11.9	5.0 ± 1.2
	EFE	980 ± 37	15.9 ± 0.8	49.1 ± 1.8	56.5 ± 7.4	1.6 ± 0.81	1.4 ± 0.5	9.8 ± 12.6	85.0 ± 13.8	3.8 ± 1.6
	Ephedra Herb	965 ± 52	15.5 ± 0.8	49.1 ± 1.4	53.1 ± 8.1	1.99 ± 0.76	2.2 ± 0.8	12.4 ± 6.6	80.6 ± 7.4	4.8 ± 2.3

* P < 0.05 vs. mice administered water, by Dunnett’s test

## Discussion

Administration of Kampo medicines containing Ephedra Herb is contraindicated for patients with hypertension or cardiomyopathies, while administration of these medicines to elderly patients or those with extreme sensitivity to Ephedra Herb requires special attention. The primary effects and adverse effects of Ephedra Herb have been traditionally believed to be mediated by ephedrine alkaloids, because ephedrine alkaloids are structurally similar to adrenaline and stimulate both sympathetic and parasympathetic neurons. However, recent data suggest that Ephedra Herb contains active ingredients other than ephedrine alkaloids, such as herbacetin glycosides [12], and possesses ephedrine alkaloid-independent pharmacological actions [13]. We hypothesized that several pharmacological actions from the herb would remain after removing the ephedrine alkaloids from it. In the present study, we show that EFE has a c-Met inhibitory action, analgesic effect, and anti-influenza activity without toxicity.

We have previously reported that Ephedra Herb suppresses HGF-induced cancer cell motility by prevention of c-Met phosphorylation via inhibition of its tyrosine kinase activity [4]. Our findings suggest that Ephedra Herb could be utilized as a novel type of c-Met inhibitor in c-Met-expressing cancer patients. However, Ephedra Herb is not suitable for cancer patients, because they do not have sufficient physical strength to tolerate its associated adverse effects. In this study, EFE exhibited efficacy as a c-Met inhibitor similar to that of Ephedra Herb extract, indicating that the c-Met inhibitory activity of Ephedra Herb is not derived from ephedrine alkaloids. Therefore, EFE or pseudo-Kampo medicines containing EFE instead of Ephedra herb could be utilized as treatments for c-Met-expressing cancer patients, because ephedrine alkaloid-induced side effects should not limit their use.

Kampo medicines containing Ephedra Herb, such as eppikajutsubuto, makyoyokukanto, kakkonto, and maoto, are used to treat myalgia, arthralgia, and rheumatism. The analgesic effects of these Kampo medicines containing Ephedra Herb are explained by the anti-inflammatory action of pseudoephedrine, a constituent of Ephedra Herb. Ephedra Herb has been reported to inhibit acute inflammation [2], and its main anti-inflammatory action is thought to be carried out by pseudoephedrine due to its inhibition of prostaglandin E2 biosynthesis [16]. However, we have recently found that herbacetin, a component of Ephedra Herb, suppressed formalin-induced pain via inhibition of NGF-TrkA signaling [17]. Formalin injection induces two different phases of pain. In the first phase of formalin-induced pain, neurogenic pain is caused by direct activation of type C fibers in nociceptive nerve endings, which release substance P, glutamine, and bradykinin, among other pain mediators. Non-steroidal anti-inflammatory agents (NSAIDs) such as aspirin and diclofenac are ineffective against the first phase of the formalin test [19, 20]. The second phase of formalin-induced pain occurs through ventral horn neuronal activation at the spinal cord level and is characterized as inflammatory pain related to the release of chemical mediators such as histamine, serotonin, bradykinin, prostaglandins, and excitatory amino acids [19, 21]. The pain associated with the second phase of the formalin test is suppressed by NSAIDs. Central analgesics, such as morphine, inhibit the pain associated with the first and second phases of the formalin test. EFE reduced the second phase of formalin-induced pain in the same manner as Ephedra Herb, suggesting that EFE acts on inflammatory pain, while indicating that the analgesic effect of Ephedra Herb is independent from pseudoephedrine. EFE could represent a novel analgesic drug without the adverse effects associated with ephedrine alkaloids. Hayashi reported that maoto, which contains Ephedra Herb, relieved bone pain associated with treatment with zoledronic acid hydrate, a therapeutic agent used to treat patients with bone lesions derived from bone cancer metastasis [22]. Therefore, EFE and pseudo-Kampo medicines containing EFE instead of Ephedra Herb may treat cancer and cancer-related pain simultaneously.

The maoto formula consists of four herbal substances: Apricot Kernel, Cinnamon Bark, Glycyrrhiza, and Ephedra Herb, the principal component. Maoto affects the early phase of influenza virus infection, and its anti-influenza activity is comparable with that of oseltamivir [23]. Furthermore, it has been reported that Ephedra Herb has an inhibitory effect on the acidification of intracellular compartments, such as endosomes and lysosomes, which inhibits the growth of influenza virus [3]. Our study revealed that EFE prevented influenza virus infection, in a manner independent of ephedrine alkaloids. EFE and a pseudo-maoto formula, consisting of the herbal substances mentioned above with EFE instead of Ephedra Herb, have none of the adverse effects associated with ephedrine alkaloids; therefore, they may be of use as therapeutic and prophylactic measures against influenza infection, especially in the elderly.

We evaluated the safety of EFE by carrying out repeated-dose toxicity studies. After 2 weeks of oral administration of Herb Ephedra, EFE, or water, there was no significant difference in the weight of any tissue, except for the colon, among the groups. The colon weights of the male mice treated with Herb Ephedra extract or EFE were significantly lower than that of the water group. However, the reduction in colon weight was small and not associated with morphological abnormalities. Furthermore, colon weight showed no significant difference between the groups of female mice. Thus, EFE has almost no effect on the colon. Neither serum biochemistry data nor hematological data showed any significant differences between mice taking EFE or water. On the other hand, there were significant differences in PLT and WBC between male mice taking Ephedra Herb extract and water. These results suggest that EFE may be safer than Ephedra Herb extract.

Ephedra Herb has an antitussive action and removes nasal obstructions by sympathomimetic effects derived from ephedrine alkaloids, but EFE is predicted to produce neither of these effects. Therefore, EFE may be unsuitable for treatment of patients with a common cold.

Until now, the pharmacological effects of Ephedra Herb were widely believed to be mediated by ephedrine alkaloids. Harada obtained alkaloid-free Ephedra Herb by selectively removing ephedrine alkaloids through ether extraction under ammonium hydroxide alkali conditions, followed by extraction with water, evaporation of the product to dryness, and addition of the product to a neutral extract, obtained by liquid–liquid partition of the ether extract. Harada reported that alkaloid-free Ephedra Herb extract did not raise blood pressure, inhibit inflammation, or reduce the severity of carrageenan-induced edema [18]; therefore, he concluded that the pharmacological actions of Ephedra Herb were due to ephedrine alkaloids. However, as noted by the author, some components in Ephedra Herb may be altered after exposure to ammonium hydroxide [18]. For example, pyran rearrangements of procyanidins have been reported at alkaline pH [24]. Moreover, Ephedra Herb has been reported to contain proanthocyanidins [7], which might be rearranged by alkaline treatment. Therefore, it is possible that alkaloid-free Ephedra Herb may lack some of the pharmacological activities of Ephedra Herb.

In this study, we demonstrated for the first time that Ephedra Herb extract does not lose its pharmacological activity after elimination of its ephedrine alkaloids. Our current objectives include identifying active substances present in the non-alkaloidal fraction of Ephedra Herb extract and obtaining licensing approval for therapeutic use of EFE.

## Electronic supplementary material

Below is the link to the electronic supplementary material.

Supplementary material 1 (TIFF 30 kb) Supplemental Fig. 1. Effect of oseltamivir on influenza virus infection in MDCK cells. MDCK cells (3 × 10^4^ cells) were incubated in 100 μl of 10 % FCS-minimal essential medium (MEM) in a 96-well plate for 24 h and washed with MEM. Next, the cells were incubated for 72 h at 37 °C in 100 μl of MEM or MEM containing a twofold serial dilution of 10 μM oseltamivir with (B) or without (A) 100 TCID_50_ of influenza virus A/WSN/33(H1N1). Next, living cells were stained with crystal violet and the absorbance (560 nm) of each sample was quantified using a microplate reader
