# Supplementary figures and images for: Ephedrine alkaloids-free Ephedra Herb extract: a safer alternative to ephedra with comparable analgesic, anticancer, and anti-influenza activities
Source: J Nat Med. 2016 Mar 4;70:571–83. doi: 10.1007/s11418-016-0979-z (PMC4935746; doi:10.1007/s11418-016-0979-z)

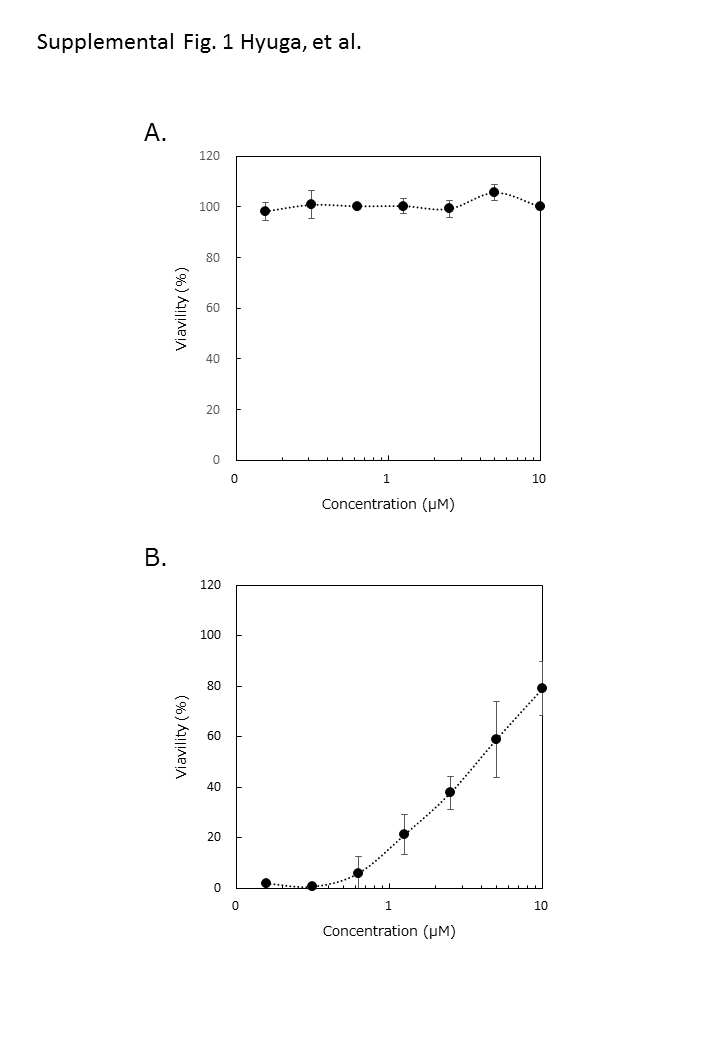

Supplement: Supplementary file 1 — Supplementary material 1 (TIFF 30 kb) Supplemental Fig. 1. Effect of oseltamivir on influenza virus infection in MDCK cells. MDCK cells (3 × 10^4^ cells) were incubated in 100 μl of 10 % FCS-minimal essential medium (MEM) in a 96-well plate for 24 h and washed with MEM. Next, the cells were incubated for 72 h at 37 °C in 100 μl of MEM or MEM containing a twofold serial dilution of 10 μM oseltamivir with (B) or without (A) 100 TCID50 of influenza virus A/WSN/33(H1N1). Next, living cells were stained with crystal violet and the absorbance (560 nm) of each sample was quantified using a microplate reader [file 11418_2016_979_MOESM1_ESM.tif]
